# Medical Lexicon: A Dictionary of Medical Science

**Published:** 1865-02

**Authors:** 


MEDICAL LEXICON. A Dictionary of Medical Science; containing a concise explanation of the various subjects and terms of Anatomy, Physiology, Pathology, Hygiene, Therapeutics, Pharmacology, Pharmacy, Surgery, Obstetrics, Medical Jurisprudence, and Dentistry; notices of Climate, and of Mineral Waters; formula for Officinal, Empirical, and Dietetic Preparations; with the Accentuation and Etymology of the Terms. And the French and other Synonymes; so as to constitute a French, as well as English, Medical Lexicon. By ROBLEY DUNGLISON, M.D., LL.D., Prof. of Institutes of Medicine, &c., in the Jefferson Medical College of Philadelphia. Thoroughly Revised and very greatly Augmented. Philadelphia: BLANCHARD & LEA. 1865.

This is a new edition of Dr. DUNGLISON'S well-known Dictionary of Medical Science. It is published in excellent style, bound in leather, and contains over 1000 closely printed pages. It is undoubtedly the most complete and useful Medical Dictionary hitherto published in this country.

For sale by W. B. KEEN & Co., 148 Lake Street, Chicago.

